# Solo, a RhoA-targeting guanine nucleotide exchange factor, is critical for hemidesmosome formation and acinar development in epithelial cells

**DOI:** 10.1371/journal.pone.0195124

**Published:** 2018-04-19

**Authors:** Sachiko Fujiwara, Tsubasa S. Matsui, Kazumasa Ohashi, Shinji Deguchi, Kensaku Mizuno

**Affiliations:** 1 Division of Bioengineering, Graduate School of Engineering Science, Osaka University, Toyonaka, Osaka, Japan; 2 Research Fellow of the Japanese Society for the Promotion of Science, Kojimachi, Chiyoda-ku, Tokyo, Japan; 3 Department of Biomolecular Sciences, Graduate School of Life Sciences, Tohoku University, Sendai, Miyagi, Japan; University of Bergen, NORWAY

## Abstract

Cell-substrate adhesions are essential for various physiological processes, including embryonic development and maintenance of organ functions. Hemidesmosomes (HDs) are multiprotein complexes that attach epithelial cells to the basement membrane. Formation and remodeling of HDs are dependent on the surrounding mechanical environment; however, the upstream signaling mechanisms are not well understood. We recently reported that Solo (also known as ARHGEF40), a guanine nucleotide exchange factor targeting RhoA, binds to keratin8/18 (K8/K18) intermediate filaments, and that their interaction is important for force-induced actin and keratin cytoskeletal reorganization. In this study, we show that Solo co-precipitates with an HD protein, β4-integrin. Co-precipitation assays revealed that the central region (amino acids 330–1057) of Solo binds to the C-terminal region (1451–1752) of β4-integrin. Knockdown of Solo significantly suppressed HD formation in MCF10A mammary epithelial cells. Similarly, knockdown of K18 or treatment with Y-27632, a specific inhibitor of Rho-associated kinase (ROCK), suppressed HD formation. As Solo knockdown or Y-27632 treatment is known to disorganize K8/K18 filaments, these results suggest that Solo is involved in HD formation by regulating K8/K18 filament organization via the RhoA-ROCK signaling pathway. We also showed that knockdown of Solo impairs acinar formation in MCF10A cells cultured in 3D Matrigel. In addition, Solo accumulated at the site of traction force generation in 2D-cultured MCF10A cells. Taken together, these results suggest that Solo plays a crucial role in HD formation and acinar development in epithelial cells by regulating mechanical force-induced RhoA activation and keratin filament organization.

## Introduction

Hemidesmosomes (HDs) are epithelial cell-specific adhesion complexes that regulate a wide range of biological processes, including cell migration, proliferation, differentiation, and apoptosis [1–3]. HDs are formed at cell-substrate adhesion sites, where α6β4-integrin binds to the extracellular matrix (ECM) on the outside of the cell, and to keratin intermediate filaments through hemidesmosomal proteins on the inside of the cell. β4-integrin interacts with plectin, which anchors keratin filaments to the hemidesmosomal adhesions [4,5]. HDs play important roles in connecting epithelial cells to the basal membrane and maintaining barrier integrity of epithelial tissues, because the loss or mutation of HD components results in epidermolysis bullosa [6].

Epithelial cells continuously perceive and respond to mechanical forces, derived from the inside and outside of the cells, which leads to reorganization of the cytoskeleton and adhesion structures to adapt to the mechanical environment [7,8]. While the stability of HDs is important for epithelial integrity, the dynamic reorganization of HDs in response to its surrounding environment is also critical for maintaining cell and tissue homeostasis. The turnover and maturation of HDs are affected not only by chemical signals, such as epidermal growth factor (EGF), but also by mechanical forces that are mediated by keratin filaments anchored to HDs [9,10]. MCF10A mammary epithelial cells cultured in 3D gels develop acinar structures, and this process depends on ECM stiffness and requires α6β4-integrin clustering and HD formation [1,11], indicating that HDs play a crucial role in sensing ECM stiffness during acinar formation in epithelial cells. However, the molecular mechanisms underlying the mechanical force-dependent control of HD organization and acinar formation remain largely unknown.

The Rho family of small GTPases are key regulators of cytoskeletal reorganization [12]. Both chemical and mechanical stimuli regulate the activities of Rho family proteins. Rho-guanine nucleotide exchange factors (Rho-GEFs) activate Rho GTPases by promoting the exchange of GDP for GTP, whereas Rho GTPase-activating proteins (Rho-GAPs) inactivate them by promoting the hydrolysis of GTP to GDP. The Dbl-related Rho-GEF family consists of 70 members and forms the largest group of Rho-GEFs in the human genome [13]. They commonly possess a pair of a catalytic Dbl homology (DH) domain and a regulatory pleckstrin homology (PH) domain, but their sequences other than the DH-PH domain are diverse [13]. Spatiotemporal regulation of the activities of Rho-GEFs and Rho-GAPs appears to be involved in the mechanical force-induced cellular responses. Indeed, multiple Rho-GEFs with distinct downstream effectors are required for the cyclic stretch-induced re-orientation of vascular endothelial cells [14]. We recently identified Solo (also known as ARHGEF40) [15,16], as a RhoA-targeting GEF that is involved in cyclic stretch-induced epithelial cell re-orientation [14]. We also showed that Solo binds to keratin-8/keratin-18 (K8/K18) filaments and that knockdown of Solo disrupts the K8/K18 network [17], indicating that Solo plays a crucial role in the formation and maintenance of K8/K18 networks.

In this study, we examined the role of Solo in HD formation. We showed that Solo associates with an HD protein, β4-integrin (β4), and that knockdown of Solo or K18, or inhibition of Rho signaling suppresses HD formation in MCF10A cells. We also showed that Solo accumulates in the region of traction force generation. Furthermore, knockdown of Solo suppressed acinar development in MCF10A cells cultured in 3D Matrigel. Our results suggest that Solo is crucially involved in HD formation through controlling RhoA signaling and keratin filament organization.

## Materials and methods

### Reagents and antibodies

Y-27632 and 4’,6-diamidino-2-phenylindol (DAPI) were purchased from Wako Pure Chemical Industries (Osaka, Japan) and Polysciences (Warrington, PA), respectively. Matrigel (growth factor-reduced) was purchased from Corning Incorporated (Corning, NY). Rabbit polyclonal antibodies against human Solo were prepared as previously described [14]. Other antibodies were purchased as follows: anti-FLAG (M2; Sigma-Aldrich, St. Louis, MO), anti-GFP (A6455; Life Technologies, Camarillo, CA), anti-β-actin (AC-15; Sigma-Aldrich), anti-K18 (DA-7; BioLegend, San Diego, CA), anti-GM130 (CSB-PA600856ESR1HU; Flarebio Biotech LLC, College Park, MD), anti-β4 (58XB4; BioLegend) for immunofluorescence, and anti-β4 (Clone 7; BD Biosciences, Franklin Lakes, NJ) for immunoblotting. Secondary antibodies, anti-mouse IgG-Alexa 488, anti-mouse IgG-Alexa 568, anti-rabbit IgG-Alexa 568, and anti-rat IgG-FITC, were purchased from Thermo Fisher Scientific (Waltham, MA).

### Plasmid construction and small interfering RNA (siRNA)

Expression plasmids encoding yellow fluorescent protein (YFP)- and FLAG-tagged Solo and deletion mutants were constructed as described previously [14,17]. To construct expression plasmids encoding β4-YFP, the cDNA encoding human β4 was amplified by polymerase chain reaction (PCR) using the MegaMam Human Transcriptome Library (Agilent Technologies, Santa Clara, CA). The YFP cDNA in the pEYFP-C1 vector (Clontech, Mountain View, CA) was replaced with the β4 cDNA, and the YFP cDNA was inserted into the C-terminal of the β4 cDNA. The plasmids encoding deletion mutants of β4 were constructed by PCR amplification, and by replacing the full-length β4 cDNA in the β4-YFP plasmid with the deleted β4 cDNA. The siRNAs targeting human Solo and K18 and the negative control siRNA were purchased from Thermo Fisher Scientific (Silencer Select siRNAs). The siRNA catolog numbers are as follows: s31287 (Solo siRNA #1), s31288 (Solo siRNA #2), s31289 (Solo siRNA #3), s7995 (K18 siRNA #1), s7996 (K18 siRNA #2), and s7997 (K18 siRNA #3).

### Cells, cell culture and transfection

COS-7 cells were obtained from RIKEN Bioresource Center and cultured in Dulbecco's modified Eagle's medium (DMEM) supplemented with 10% fetal bovine serum (FBS). MCF10A cells were obtained from ATCC and cultured in DMEM/Ham's F-12 media supplemented with 5% horse serum, 20 ng/mL epidermal growth factor (EGF), 0.5 mg/mL hydrocortisone, 100 ng/mL cholera toxin, 10 μg/mL insulin, and penicillin/streptomycin. The 3-D on-top culture of MCF10A cells was performed, according to a previously described procedure [18], except that we used φ15-mm coverslips and 24-well culture dishes, but not 8-well chamber slides. Briefly, the cells were seeded onto a solidified layer of Matrigel and cultured in DMEM/Ham's F-12 supplemented with 2% horse serum, 5 ng/mL EGF, 0.5 mg/mL hydrocortisone, 100 ng/mL cholera toxin, 10 μg/mL insulin, and 2% Matrigel. Media were replaced every fourth day. Plasmid DNAs were transfected using jetPEI DNA transfection reagent (Polyplus transfection; Illkirch-Graffenstaden, France). siRNAs were transfected into cells using Lipofectamine RNAiMAX (Life Technologies). The siRNAs were used at a final concentration of 5 nM.

### Co-immunoprecipitation assay

For analyzing the interaction between YFP-Solo and β4, MCF10A cells were transfected with YFP-Solo or its mutants and lysed with ice-cold lysis buffer [1% NP-40, 5% glycerol, 50 mM Tris-HCl (pH 7.5), 150 mM NaCl, 0.5 mM EGTA, 50 mM NaF, 1 mM Na_3_VO_4_, 1 mM DTT, 0.25 μM PMSF, 10 μg/mL leupeptin, and 2 μg/mL pepstatin]. Cell lysates were clarified by centrifugation at 18,000 × *g* for 10 min and incubated for 2 h at 4°C with an anti-GFP antibody. Immunoprecipitates were analyzed by immunoblotting with an anti-β4 antibody. For mapping the Solo-binding region of β4, COS-7 cells were co-transfected with FLAG-Solo and deletion mutants of β4-YFP and lysed with ice-cold lysis buffer. Cell lysates were clarified and incubated for 2 h at 4°C with an anti-FLAG antibody. Immunoprecipitates were analyzed by SDS-PAGE, followed by immunoblotting with anti-FLAG and anti-GFP antibodies. For mapping the β4-binding region of Solo, β4-YFP (1451–1752) was co-transfected with FLAG-Solo or its deletion mutants, and the cell lysates were analyzed by immunoprecipitation with an anti-FLAG antibody, followed by immunoblotting with anti-FLAG and anti-GFP antibodies. Immunoblotting were conducted under the following conditions: Blocking, 5% skimmed milk-containing TBS-T (0.1% Tween-20-containing Tris-buffered saline) for 1 h at room temperature; primary antibody incubation, 5% BSA-containing TBS-T for overnight at 4°C; secondary antibody incubation, 5% skimmed milk-containing TBS-T for 40 min at room temperature.

### Immunostaining and fluorescence imaging

For visualizing the HDs of 2D-cultured cells, MCF10A cells were seeded onto a coverslip coated with 3% v/v Matrigel diluted with DMEM/Ham's F-12. Cells were cultured with growth medium for 48 h and then starved with starvation medium (DMEM/Ham's F-12 media supplemented with 0.2% horse serum, 0.5 mg/mL hydrocortisone, 100 ng/mL cholera toxin, 10 μg/mL insulin, and penicillin/streptomycin) overnight. Cells were fixed with 100% methanol at –20°C for 10 min and blocked with 2% FBS in phosphate-buffered saline (PBS). Cells were then incubated with an anti-β4 antibody in Can Get Signal immunostain solution (Toyobo, Osaka, Japan) at 4°C overnight. Anti-mouse IgG-Alexa 568 was used as a secondary antibody. After washing with PBS, stained cells were treated with 4% paraformaldehyde at 20°C for 10 min before mounting. For acinar staining of 3D-cultured MCF10A cells, cells were fixed with 4% paraformaldehyde in PBS at room temperature for 30 min, permeabilized with 0.5% Triton X-100 in PBS at 4°C for 10 min, and blocked with 10% FBS in the blocking buffer (130 mM NaCl, 7 mM Na_2_HPO_4_, 3.5 mM NaH_2_PO_4_, 0.1% bovine serum albumin, 0.2% TritonX-100, 0.05% Tween-20, and 5% FBS) at 20°C for 40 min. Cells were then incubated with appropriate antibodies in the blocking buffer at 4°C overnight. Anti-rat IgG-FITC, anti-mouse IgG-Alexa 488, anti-mouse IgG-Alexa 568, and anti-rabbit IgG-Alexa 568 were used as secondary antibodies, and DAPI was used to stain the nuclei. Fluorescent images were obtained using a laser scanning confocal microscope LSM 710 (Carl Zeiss, Jena, Germany), equipped with a PL-Apo 63× oil objective lens (NA 1.4). Images were analyzed using ImageJ software.

### Wrinkle formation assay

Cell traction force was visualized using a wrinkle generating method as described previously [19], except that silicone substrates CY 52–276 (Dow Corning Toray, Tokyo, Japan) were mixed at a weight ratio of 1.2:1 and the substrates were coated with 3% v/v Matrigel diluted with DMEM/Ham's F-12. For time-lapse observation of wrinkle formation, the dishes were placed at 37°C in an atmosphere of 5% CO_2_ using a stage incubator (Tokai Hit, Shizuoka, Japan), and phase-contrast and fluorescent images were taken every 5 min with an IX71 inverted microscope (Olympus, Tokyo, Japan) equipped with a 40× objective lens (NA 0.6). Confocal images were taken with an FV1000 microscope (Olympus) equipped with a 60× objective lens (NA 1.4).

### Statistical analysis

Data are expressed as the means ± standard deviation (SD) of more than three independent experiments. Statistical analysis was performed using Prism 7 (GraphPad Software). *p*-values were calculated using a one-way analysis of variance (ANOVA) followed by Dunnett's test or a two-tailed paired *t*-test. Statistical significance was set at *P* < 0.05.

## Results

### Solo interacts with β4-integrin

**Fig 1:**
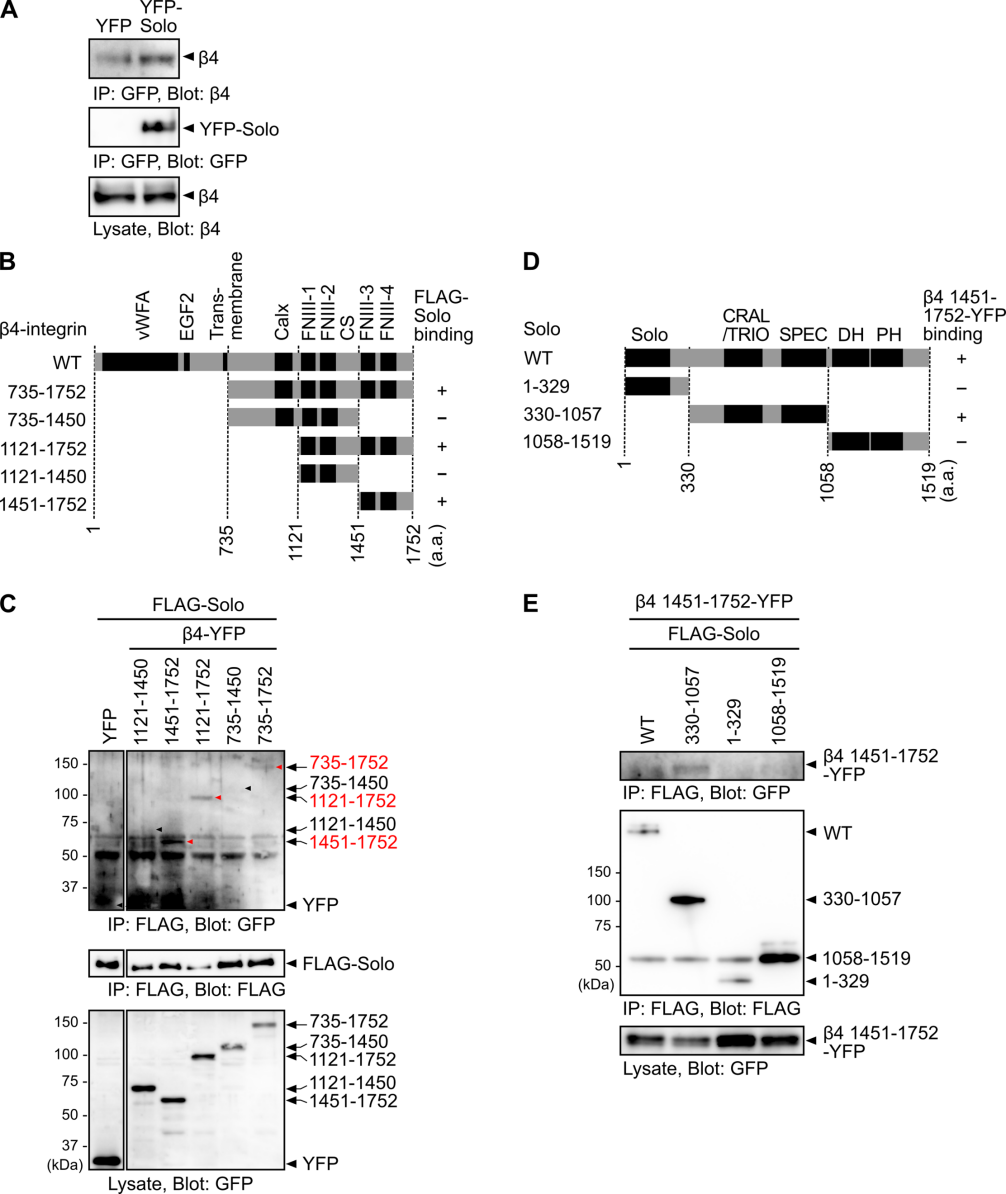
Solo binds to β4-integrin. (A) Co-immunoprecipitation assays. YFP-Solo was expressed in MCF10A cells and the cell lysates were immunoprecipitated (IP) with an anti-GFP antibody and analyzed by immunoblotting with anti-GFP and anti-β4 antibodies. (B–E) Mapping of the binding regions of Solo and β4. (B) Schematic domain structure of β4 and its deletion mutants used in this study. Numbers denote amino acid residues flanking each region. The binding ability of each fragment to FLAG-Solo is indicated in the right column. Conserved domains are denoted as: vWFA, von Willebrand factor type A; EGF, EGF-like; Calx, Calx-beta; FNIII, fibronectin type III; CS, connecting segment. (C) Co-immunoprecipitation assays of β4 fragments with Solo. YFP-tagged β4 fragment (β4-YFP) and FLAG-tagged Solo-WT were co-expressed in COS-7 cells, and the cell lysates were immunoprecipitated with an anti-FLAG antibody and analyzed by immunoblotting with anti-FLAG and anti-GFP antibodies. Arrowheads indicate the expected positions of YFP-tagged β4 fragments. (D) Schematic domain structure of Solo and its deletion mutants used in this study. The binding ability of each fragment to β4 (1451–1752)-YFP is indicated in the right column. Conserved domains are indicated as Solo, CRAL/TRIO, SPEC (spectrin repeats), DH, and PH domains. (E) Co-immunoprecipitation assays of Solo fragments with β4. FLAG-Solo or its fragments were co-expressed with β4 (1451–1752)-YFP in COS-7 cells, and the cell lysates were immunoprecipitated with an anti-FLAG antibody and analyzed by immunoblotting with anti-FLAG and anti-GFP antibodies. (A, C, and E) These experiments were repeated more than three times and reproducible results were obtained.

To investigate the role of Solo in HD regulation, we first analyzed whether Solo interacts with the hemidesmosomal protein, β4-integrin (β4). YFP-tagged Solo was expressed in MCF10A human mammary epithelial cells, and the binding ability of Solo to endogenous β4 was analyzed by immunoprecipitation with an anti-GFP antibody, followed by immunoblotting with an anti-β4 antibody. Endogenous β4 was co-precipitated with YFP-Solo (Fig 1A), indicating that Solo has the potential to bind to β4-integrin.

### Mapping of the binding regions of β4 and Solo

β4 possesses an unusually long cytoplasmic region, which contains a Calx-β domain, two tandemly aligned pairs of fibronectin type III (FNIII) domains (FNIII-1/2 and FNIII-3/4) that are separated by the connecting segment (CS), and the C-terminal tail region [20,21]. To determine which region of β4 is involved in Solo binding, YFP-tagged cytoplasmic fragments of β4 were constructed (Fig 1B), and they were individually co-expressed with FLAG-tagged Solo in COS-7 cells. Cell lysates were immunoprecipitated with an anti-FLAG antibody, and the precipitates were analyzed by immunoblotting with an anti-GFP antibody. YFP-tagged β4 fragments (735–1752, 1121–1752, and 1451–1752) were co-precipitated with FLAG-Solo, but other fragments (735–1450 and 1121–1450) were not (Fig 1C). These results indicate that β4 binds to Solo through the C-terminal region (1451–1752), consisting of FNIII-3/4 domain and the C-terminal tail.

Solo has several conserved domains, including a Solo domain, a CRAL/TRIO domain, spectrin repeats, a DH domain, and a PH domain [17]. To map the β4-binding region of Solo, FLAG-tagged Solo and its deletion mutants were constructed (Fig 1D) and individually co-expressed with YFP-tagged β4 fragment 1451–1752 in COS-7 cells. Cell lysates were immunoprecipitated with an anti-FLAG antibody, and the precipitates were analyzed by immunoblotting with an anti-GFP antibody. YFP-tagged β4 fragment 1451–1752 was co-precipitated with FLAG-Solo-WT and its fragment 330–1057, but not with 1–329 or 1058–1519 (Fig 1E), indicating that Solo binds to β4 through the central region containing a CRAL/TRIO domain and spectrin repeats.

### Knockdown of Solo suppresses HD formation

**Fig 2:**
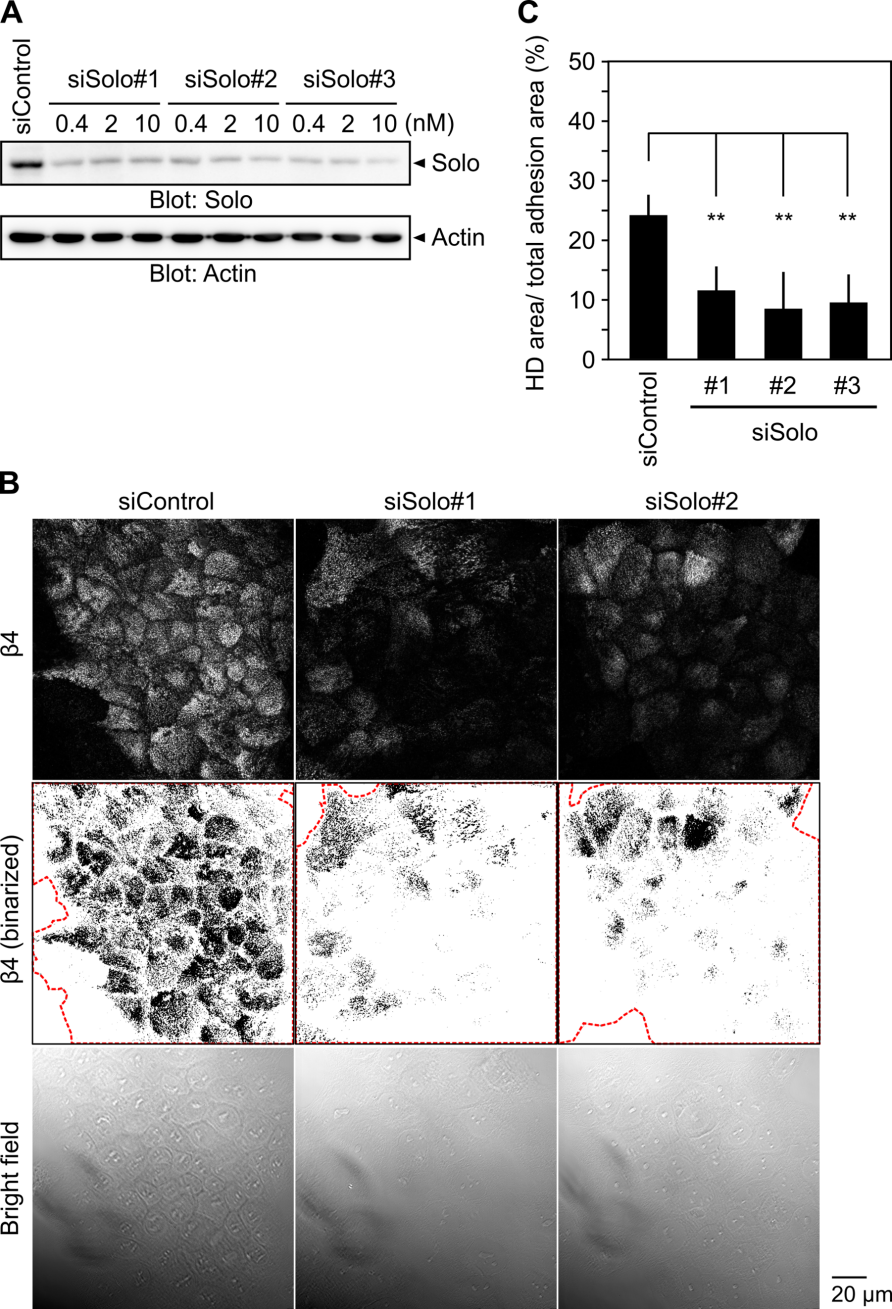
Knockdown of Solo suppresses hemidesmosome formation. (A) Effects of Solo-targeting siRNAs on Solo expression. MCF10A cells were transfected with control or Solo-targeting siRNAs at the indicated concentrations of siRNAs and cultured for 48 h. Cell lysates were analyzed by immunoblotting with an anti-Solo antibody. (B) Ventral images of endogenous β4, their binary images, and bright field images of control and Solo knockdown MCF10A cells. Cells were seeded on a thin Matrigel-coated coverslip, transfected with control or Solo-targeting siRNAs, and cultured for 48 h. The red dotted lines indicate the total adhesion area defined by bright field images. Scale bar, 20 μm. (C) Quantitative analysis of the effect of Solo knockdown on HD formation. The ratio of HD area (measured from the binary image of β4) to total adhesion area (measured from the bright field image) was calculated. Data represent the means ± SD of 4 independent experiments (at least 5 images per experiment). **P < 0.01 (one-way ANOVA followed by Dunnett's test).

To examine whether Solo is involved in HD formation, we analyzed the effect of Solo knockdown on HD formation. Immunoblot analysis revealed that three independent siRNAs targeting Solo effectively suppress the expression of endogenous Solo in MCF10A cells (Fig 2A). MCF10A cells are known to form HD structures when they are monolayer-cultured on laminin-enriched matrix and starved for growth factors [22]. To induce HD formation at high frequency, MCF10A cells were cultured on coverslips coated with thin Matrigel and starved for serum and EGF for 12 h before fixation. HDs were visualized by immunostaining with a β4-specific antibody. Because β4 is predominantly located in HDs in epithelial cells [23], we defined the β4-positive area as the HD area of the cells. The β4 signals were binarized and the β4-positive area was calculated using ImageJ software. The total adhesion area of the cells was measured by a bright field microscopy. To quantify the effect of Solo knockdown on HD formation, the percentage of the β4-positive area in the total cell adhesion area was calculated. In control siRNA-treated cells, β4-positive signals were strongly and widely distributed over the cell adhesion region (Fig 2B). Confocal microscopic analysis of the slice images of β4 showed the predominant localization of β4 near the ventral surface of the cells (Panel A in S1 Fig). Knockdown of Solo had no apparent effect on the total adhesion area of the cells, but significantly decreased the ratio of the β4-positive area to the total cell adhesion area (Fig 2B and 2C). Immunoblot analysis revealed that Solo knockdown has no apparent effect on the expression level of endogenous β4 (Panel B in S1 Fig). These results indicate that knockdown of Solo did not reduce the expression level of β4, but suppressed its localization to HDs.

### Treatment with Y-27632 suppresses HD formation

**Fig 3:**
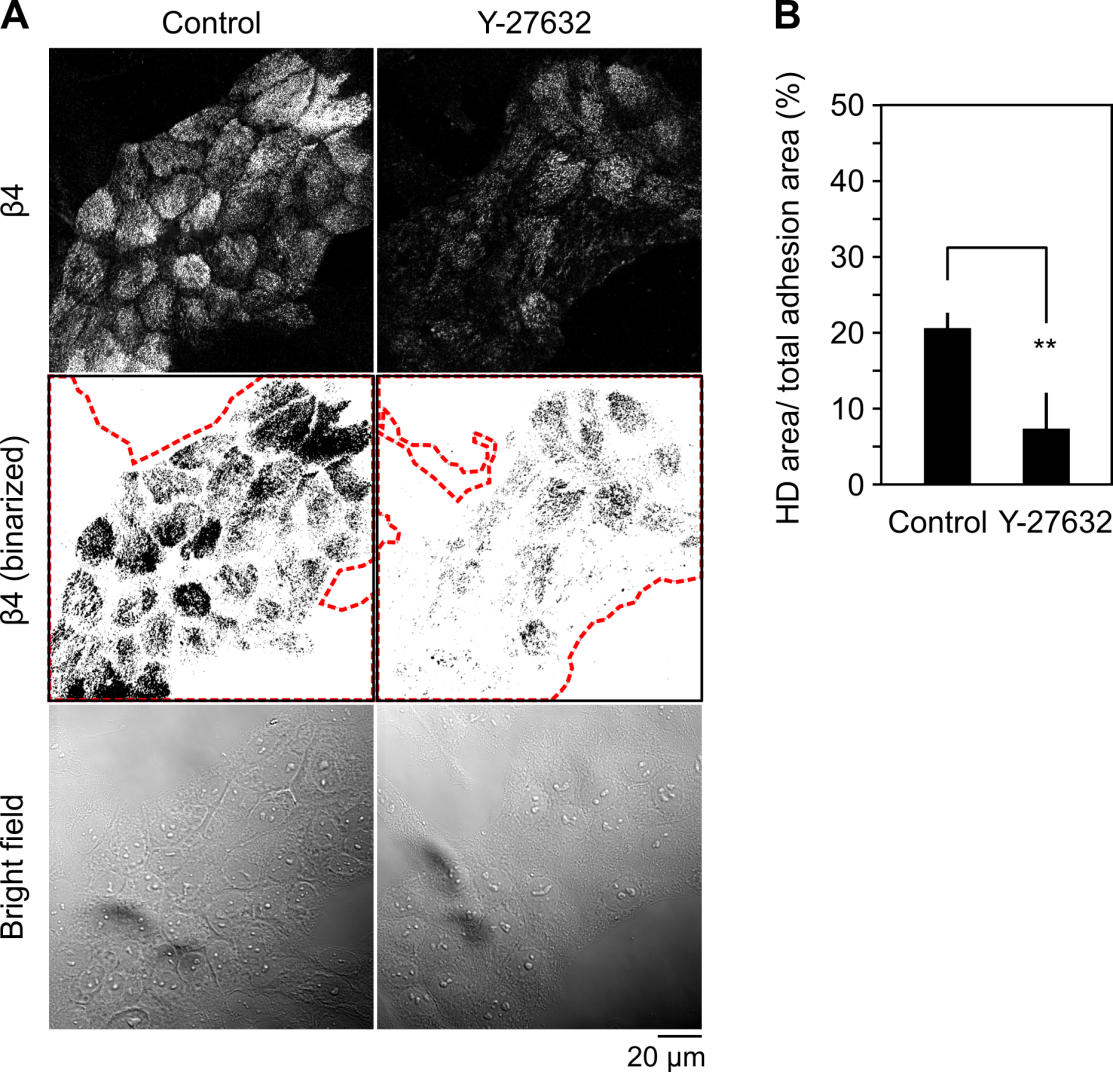
ROCK inhibitor suppresses hemidesmosome formation. (A) Ventral images of endogenous β4, their binary images, and bright field images of Y-27632-treated or untreated MCF10A cells. Cells were cultured as shown in Fig 2 and treated with 10 μM of Y-27632 or left untreated for 24 h. The red dotted lines indicate the total adhesion area defined by the bright field images. Scale bar, 20 μm. (B) Quantitative analysis of the effect of Y-27632 on HD formation. The ratio of HD area to total adhesion area was calculated, as in Fig 2. Data represent the means ± SD of 4 independent experiments (at least 7 images per experiment). **P < 0.01 (two-tailed paired *t*-test).

Because Solo is a RhoA-targeting GEF, we next examined whether the RhoA-ROCK pathway is involved in HD formation, using Y-27632, a selective inhibitor of ROCK. MCF10A cells were cultured on a Matrigel-coated coverslip and treated with 10 μM Y-27632 for 24 h under serum-starved conditions. Cells were then fixed and stained with an anti-β4 antibody to analyze HD formation. Treatment with Y-27632 for 24 h had no apparent effect on the total adhesion area, but significantly decreased the ratio of the β4-positive area to the total cell adhesion area, indicating that the Rho-ROCK pathway is required for HD formation (Fig 3A and 3B).

### Knockdown of K18 suppresses HD formation

**Fig 4:**
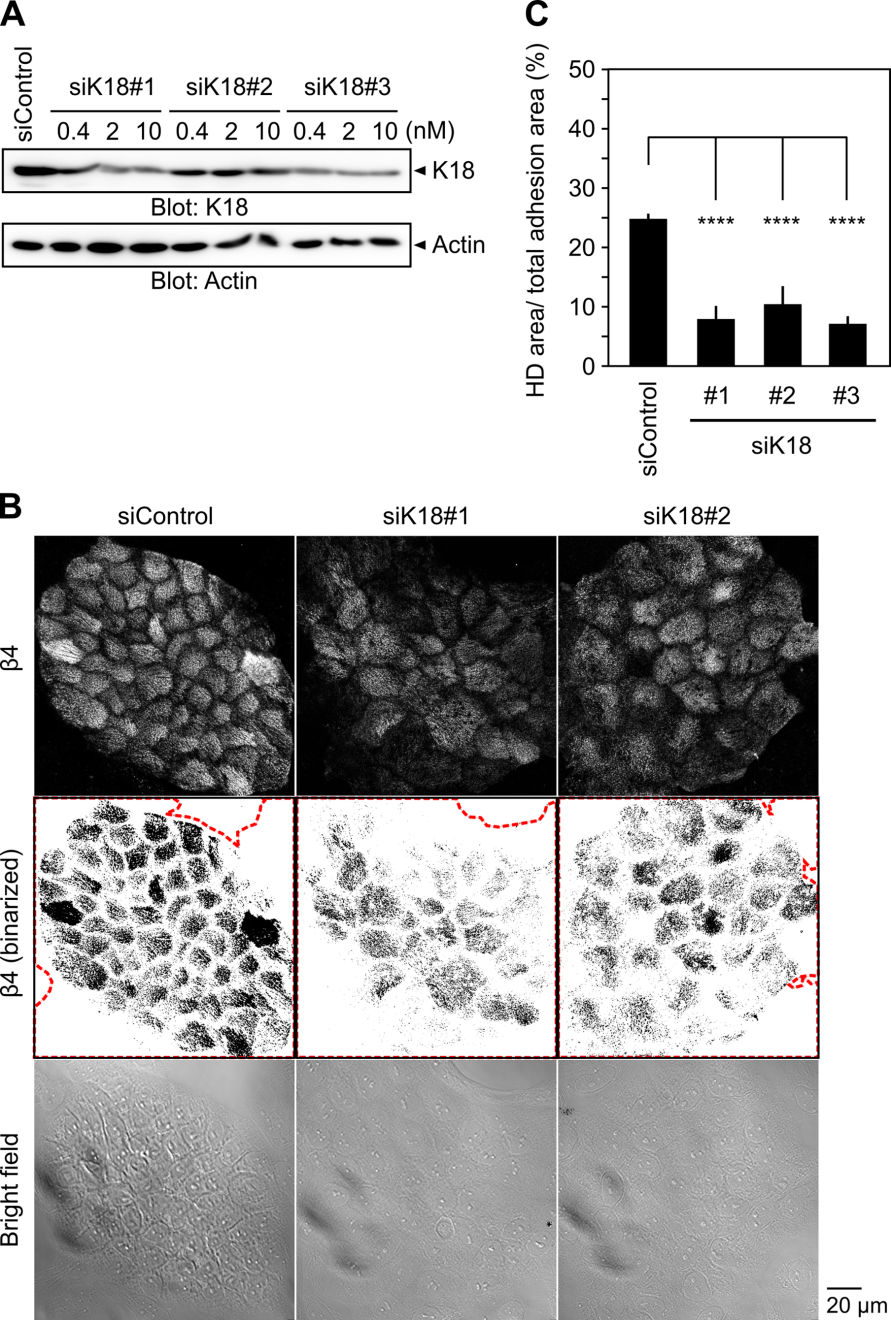
Knockdown of keratin-18 suppresses hemidesmosome formation. (A) Effects of K18-targeting siRNAs on K18 expression. MCF10A cells were transfected with control or K18-targeting siRNAs at the indicated concentrations of siRNAs and cultured for 48 h. Cell lysates were analyzed by immunoblotting with an anti-K18 antibody. (B) Ventral images of endogenous β4, their binary images, and bright field images of control and K18 knockdown MCF10A cells. Cells were seeded as shown in Fig 2, transfected with control or K18-targeting siRNAs, and cultured for 48 h. The red dotted lines indicate the total adhesion area defined by bright field images. Scale bar, 20 μm. (C) Quantitative analysis of the effect of K18 knockdown on HD formation. The ratio of HD area to total adhesion area was calculated, as in Fig 2. Data represent the means ± SD of 3 or 4 independent experiments (at least 7 images per experiment). ****P < 0.0001 (one-way ANOVA followed by Dunnett's test).

Keratin filaments play an essential role in regulating the stability of HDs at cell-substrate adhesion sites [24,25]. We recently showed that knockdown of Solo causes disorganization of K8/K18 filaments [17]. To examine the role of K8/K18 filaments in HD formation, we analyzed the effect of K18 knockdown on HD formation. siRNAs targeting K18 effectively suppressed the expression of endogenous K18 in MCF10A cells (Fig 4A). MCF10A cells were treated with K18-targeting siRNAs and the effect of K18 knockdown on HD formation was analyzed by measuring the ratio of the β4-positive area to the total cell adhesion area, as described above. K18 knockdown had no apparent effect on the total adhesion area of the cells, but significantly decreased the ratio of the β4-positive area to the total cell adhesion area (Fig 4B and 4C). Immunoblot analysis revealed that the expression level of β4 did not change after treatment with K18 siRNAs (Panel C in S1 Fig). These results indicate that K8/K18 filaments are not involved in regulating the expression level of β4, but are required for the localization of β4 to HDs.

### Knockdown of Solo impairs acinar development

**Fig 5:**
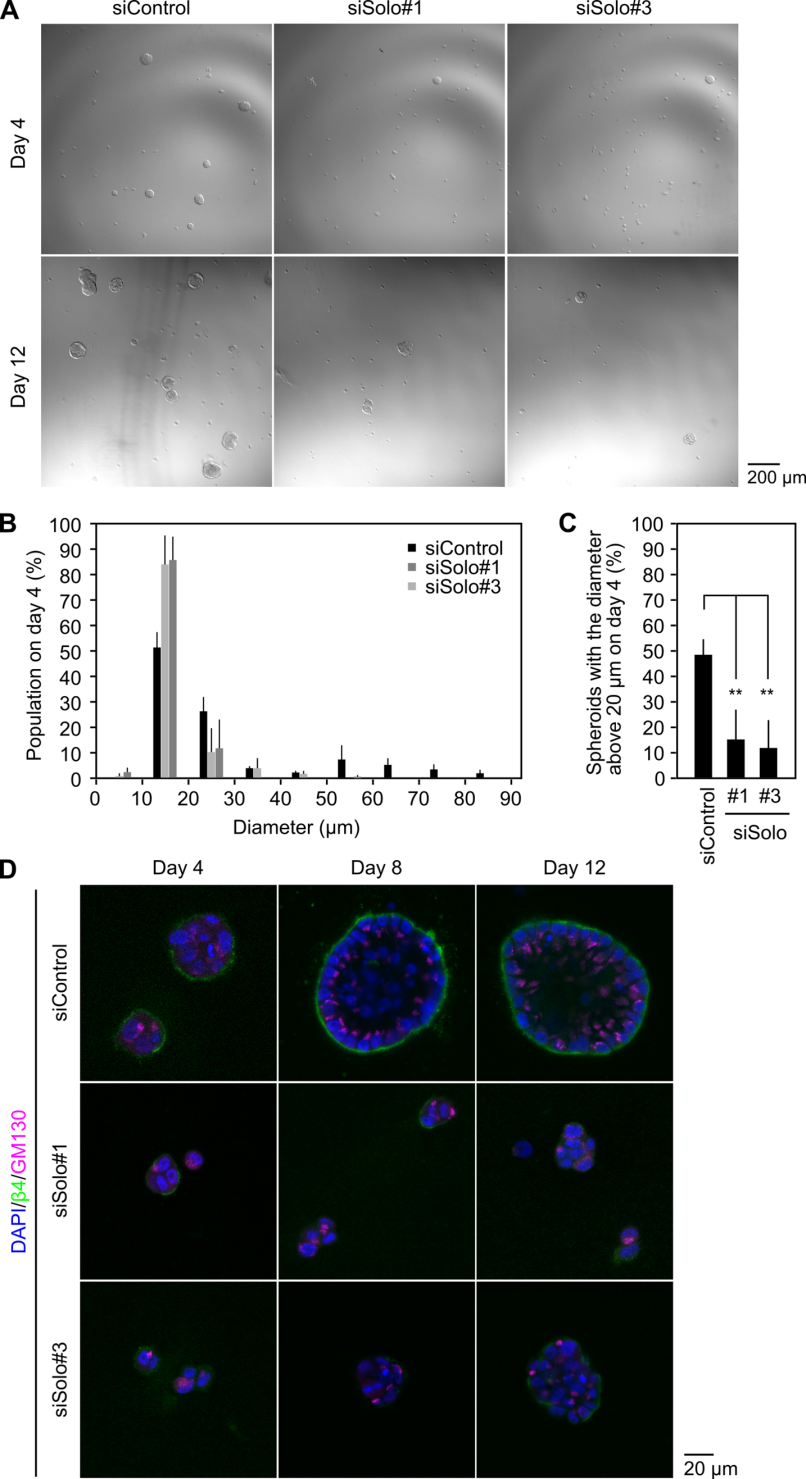
Knockdown of Solo impairs acinar development in 3D-cultured MCF10A cells. (A) Bright field images of MCF10A acini. MCF10A cells transfected with control of Solo-targeting siRNAs were seeded onto a solidified layer of Matrigel and allowed to grow in a medium containing 2% Matrigel for 4 and 12 days. Scale bar, 200 μm. (B) Size distributions of MCF10A cell clusters cultured for 4 days. Acini diameters were measured by ImageJ software. (C) Quantitative analysis of the effect of Solo knockdown on acinar growth. The percentage of cell clusters with a diameter above 20 μm was calculated. Data represent the means ± SD of 3 independent experiments. **P < 0.01 (one-way ANOVA followed by Dunnett's test). (D) Representative slice images of 3D-cultured MCF10A cells acquired with a confocal microscope. MCF10A cells were grown in a 3D culture system and cultured for 4, 8, or 12 days. Cells were fixed and stained with antibodies against β4 (green) and GM130 (magenta). GM130 was used as an apical polarity marker. The experiments were repeated more than three times for each time points and reproducible results were obtained. Scale bar, 20 μm.

MCF10A cells form polarized acinar structures when cultured in 3D Matrigel. The 3D culture system of MCF10A cells has been used as an in vitro model to investigate the mechanisms of breast tissue development and tumorigenesis [26,27]. Although MCF10A cells do not develop tight junctions, they establish and maintain the apico-basal polarity, as observed by the apical orientation of the Golgi apparatus. In the early stage of acinar development, HDs are formed at the adhesion sites facing the laminin-rich ECM surrounding the cell cluster. Since Solo is involved in HD formation, we next examined whether knockdown of Solo affects acinar formation in MCF10A cells. Cells were cultured in 3D Matrigel as follows: cells were suspended and loaded onto the stiff Matrigel and then cultured in the growth medium containing 2% Matrigel. Around 50% of the control cells formed spheroids with a diameter of more than 20 μm by day 4 in 3D culture, and then further developed acini by day 12 (Fig 5A). In contrast, Solo knockdown cells rarely formed spheroids and remained as single cells or formed a cluster of few cells with a diameter less than 20 μm by day 12 (Fig 5A). Quantitative analysis revealed that knockdown of Solo significantly decreased the frequency of formation of spheroids with a diameter of more than 20 μm by day 4 (Fig 5B and 5C). Knockdown of Solo also significantly suppressed proliferation of MCF10A cells in 2D culture (S2 Fig), indicating that Solo is required for proliferation of MCF10A cells in both 2D and 3D culture conditions.

Acinar morphogenesis is a multistep process that involves proliferation, polarization, inner cell death, and lumen formation [28]. To investigate the role of Solo in acinar development, MCF10A cells were treated with control or Solo-targeting siRNAs and 3D-cultured for 4, 8, and 12 days, and the states of acinar development and polarization were analyzed by immunostaining with antibodies against β4 (a marker of HDs) and GM130 (a marker of *cis*-Golgi). Confocal microscopic analysis of the equatorial images of cell clusters revealed that control siRNA-treated cells grew to form a cell cluster by day 4, and developed the acinar structure enclosing a hollow lumen by day 12. Polarized localization of β4 at the basal layer and GM130 at the apical side was established by day 4, and maintained during acinar development until day 12 (Fig 5D). By contrast, Solo knockdown cells exhibited substantially delayed cell proliferation and failed to develop the acinar structure. In the cluster of Solo knockdown cells, β4 was only weakly localized or unlocalized to the basal surface, and GM130 often mislocalized to the basal or lateral side of the cells (Fig 5D). These results suggest that knockdown of Solo inhibits cell proliferation, disrupts HD formation and apico-basal polarity formation, and thereby impairs acinar development.

### Solo localizes at the sites of traction force generation

**Fig 6:**
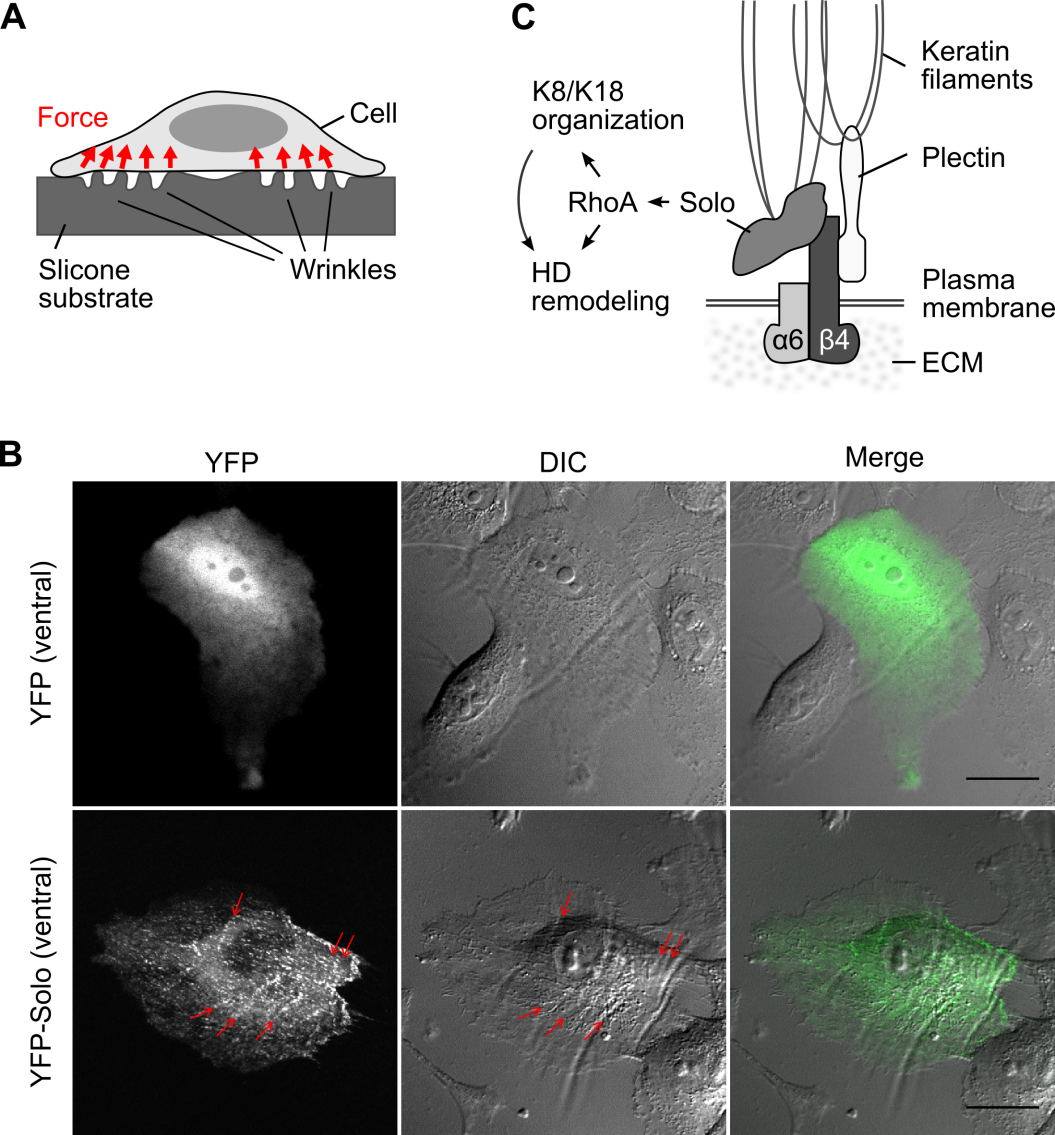
Localization of Solo at the sites of traction force generation and a model for the role of Solo in hemidesmosome remodeling. (A) Schematic illustration of the side view of the cell on silicone substrates. Wrinkles appear on the substrate depending on the forces exerted by the cells. (B) Wrinkle formation assay. MCF10A cells were transfected with YFP or YFP-Solo, seeded on a thin Matrigel-coated silicone substrate, and cultured for 24 h. Ventral images of YFP (green) and phase-contrast images were acquired with a confocal microscopy. Red arrows indicate ventral localization of Solo, particularly along the wrinkles. Scale bar, 20 μm. (C) A model for Solo-mediated HD remodeling. Solo localizes at the site of force generation on the ventral surface of epithelial cells and promotes HD formation by activating RhoA signaling and reorganizing keratin networks.

Previous studies showed that HD formation and polarized acinar development are regulated by surrounding mechanical environment (such as ECM stiffness) and cellular traction forces at cell-substrate adhesions [10,11]. To examine the involvement of Solo in sensing and transducing mechanical signals, we next investigated the locational relationship between Solo and traction forces. The traction forces in MCF10A cells were analyzed by using silicone elastomer dishes that can visualize traction forces as wrinkles on the substrate (Fig 6A) [19,29]. To apply this wrinkle formation assay for localization analysis, we acquired detailed measurement data on the wrinkles using an atomic force microscopy (AFM) and found that the height of wrinkles formed by individual cells ranges 200–700 nm (Panel A in S3 Fig). This result suggests that the existence of wrinkles can be detected by confocal microscopic analysis, but a slight displacement of z-position cannot be allowed for chasing the wrinkles in time-lapse analysis, given that the height of wrinkles is comparable to or smaller than the vertical (z-direction) resolution of confocal microscopy, i.e., 500–700 nm [30]. Based on these results, we used a confocal microscopy for analyzing the locational relationship between Solo and wrinkles and an epifluorescence microscopy for time-lapse analysis. MCF10A cells were cultured on a Matrigel-coated silicone elastomer dish, transfected with YFP or YFP-tagged Solo, and incubated overnight under serum- and EGF-starved conditions to induce cell-substrate adhesions. Confocal microscopic analysis revealed that YFP was diffusely distributed in the cytoplasm and nucleus, but YFP-Solo localized to punctate dots along the wrinkles on the ventral surface of the cells (Fig 6B). Time-lapse analysis showed that YFP-Solo, but not YFP, localized in the vicinity of the wrinkles and moved with the locomotion of wrinkles (Panel B in S3 Fig, S1 and S2 Videos). These results suggest that Solo is localized to the force-generating sites and is likely involved in sensing the mechanical environment at the cell-ECM adhesion sites.

## Discussion

In this study, we showed that Solo interacts with β4-integrin. Co-precipitation assays revealed that Solo binds to the C-terminal region (consisting of FNIII-3/4 domain and the C-terminal tail) of β4. As plectin binds to the region consisting of FNIII-1/2 domain and the N-terminal portion of the CS [21,31], Solo and plectin bind to distinct regions of the intracellular domain of β4. Because Solo and plectin also bind to keratin filaments [17,32], both of these proteins appear to function as linker proteins to anchor keratin filaments to β4 in HDs (Fig 6D). Structural studies proposed a model that plectin binding induces conformational changes of the CS region of β4, which promotes the binding of the HD proteins, BP180 and BP230, to the FNIII-3/4 domain of β4 and their recruitment to HDs and facilitates HD maturation [21,33]. Since Solo binds to the C-terminal FNIII-3/4-containing region of β4, it is possible to consider that Solo recruitment to HDs is regulated by plectin binding to β4. Further studies are required to understand the mechanism of Solo recruitment to HDs and the role of Solo-β4 interaction in HD maturation.

We showed that knockdown of Solo or K18 significantly suppressed HD formation in 2D-cultured MCF10A cells. A previous study also showed that knockout of all keratin species leads to scattering of HD proteins from the cell-substrate adhesion zone in keratinocytes, indicating that keratin filaments are essential for HD stabilization [24]. Since Solo is required for the proper organization of keratin networks in epithelial cells [17], Solo probably contributes to HD formation through regulating the keratin network organization. Considering that Solo is a GEF for RhoA and that treatment of cells with Y-27632, a specific inhibitor of ROCK, causes disorganization of K8/K18 filaments [17] and suppression of HD formation (in this study), it is likely that the Solo regulates the organization of keratin filaments and HDs through the RhoA-ROCK pathway (Fig 6D). The molecular mechanisms of how the Rho family of GTPases regulate keratin and HD organization are not well understood. In *C*. *elegans*, CED-10 (a homologue of Rac) is involved in tensional force-induced maturation of HDs that are formed between the epidermis and the body-wall muscles. Tension induces recruitment of the adapter protein GIT-1 to HDs and stimulates PAK-1 activity through PIX-1 and Rac, leading to phosphorylation of intermediate filaments to induce HD maturation [9]. However, the mechanism by which RhoA regulates keratin and HD organization remains unknown. Our observations indicate that Solo is involved in keratin network organization and HD formation and/or maintenance by regulating the RhoA-ROCK signaling pathway (Fig 6D), but further studies are required for better understanding of the mechanism of RhoA-induced reorganization of keratin filaments and HDs.

Mechanical inputs to cell-substrate adhesion sites are important for HD remodeling [10,33], but the molecular nature of mechanosensors in HDs remains poorly understood. Solo is required for force-induced RhoA activation and stress fiber formation in epithelial cells [17]. In this study, we provide evidence that Solo localizes and moves simultaneously with the traction force-generating sites at the cell-substrate adhesion interfaces, where cells sense their mechanical environment. In addition, Solo binds to both K8/K18 filaments and β4. Taking into account these observations, it is conceivable that Solo is activated by conformational change, mediated by tensional force between keratin filaments and β4 at cell-substrate adhesion sites. It is also possible that tensional force leads to Solo activation by inducing Solo phosphorylation or association with proteins that activate Solo. Further studies are required to understand the mechanism of tensional force-induced Solo activation.

MCF10A cells undergo a series of cellular events to form acinar structures, when cultured in 3D laminin-rich Matrigel [26]. Acinar morphogenesis starts from cell proliferation, followed by polarization and lumen formation. A previous study showed that expression of an inactive mutant of β4, which lacks the cytoplasmic tail, suppresses HD formation, and impairs cell polarization and acinar formation [1], indicating that β4-mediated HD formation at cell adhesion sites facing the surrounding laminin-rich Matrigel is essential for cells to acquire the apico-basal polarity and form acinar structures. In this study, we showed that knockdown of Solo markedly decreased the efficiency of acinar formation in 3D-cultured MCF10A cells. Knockdown of Solo also caused a defect in β4 accumulation at the border between the cells and ECM, and impaired the apico-basal polarity of the cell cluster. These results suggest that Solo serves to develop polarized acinar structures by promoting HD formation in response to the mechanical environment. Solo knockdown also significantly suppressed cell proliferation, probably due to the reduced activity of RhoA [34,35]. Thus, it seems likely that Solo is involved in acinar formation by regulating HD formation, cell polarity formation, and cell proliferation. Further studies are needed to understand the molecular mechanisms of Solo-mediated acinar morphogenesis and the physiological and pathological roles of Solo in tissue development and homeostasis.

## Supporting information

S1 Fig Effects of knockdown of Solo or K18 on the localization and the expression level of β4. (A) Effect of Solo knockdown on the ventral localization of β4. MCF10A cells were seeded on a thin Matrigel-coated coverslip, transfected with control or Solo-targeting siRNAs, and cultured for 48 h. Cells were then fixed and permeabilized with cold methanol and β4 was stained. The slice image was obtained at the ventral surface where the most abundant and clearest β4 signals were observed, and the additional images were taken every 0.5 μm to the dorsal side (2 slices) and to the ventral side (1 slice) using a confocal microscopy. Scale bar, 20 μm. (B and C) Effect of Solo knockdown (B) and effect of K18 knockdown (C) on the β4 protein expression level. MCF10A cells were transfected with control siRNA or Solo- or K18-targeting siRNAs and cultured for 48 h. Cell lysates were analyzed by immunoblotting with indicated antibodies. Actin was used as a loading control.
(TIF)

S2 Fig Effect of Solo knockdown on MCF10A cell proliferation. MCF10A cells were transfected with control or Solo-targeting siRNAs, seeded on 35-mm dishes, and then collected. The cell number at indicated days was calculated. Data represent the means ± SD of 3 independent experiments. **P < 0.01 (one-way ANOVA followed by Dunnett's test); n.s., not significant.
(TIF)

S3 Fig Time-lapse observation of wrinkle formation and YFP localization. (A) Detailed measurement of the wrinkles on the silicone substrate. Wrinkles generated by a single cell were simultaneously observed by phase-contrast and atomic force microscopies to evaluate the height of the wrinkles along line (i)-(ii). Scale bar, 20 μm. (B) Wrinkle formation assay. MCF10A cells were transfected with YFP or YFP-Solo, seeded on a thin Matrigel-coated silicone substrate, and cultured for 24 h. Time-lapse fluorescence images of YFP (green) and phase-contrast images were acquired every 5 min for 2.5 h (see Supplemental S1 and S2 Videos). Red arrowheads indicate accumulation of Solo along the wrinkles. Scale bar, 20 μm.
(TIF)

S1 Video Time-lapse observation of wrinkle formation and YFP localization. MCF10A cells were transfected with YFP and cultured on a thin Matrigel-coated silicone substrate for 24 h. Frames were acquired every 5 min for 2.5 h and are displayed at 4 frames/s. Scale bar, 20 μm. Related to S3A Fig, YFP.
(AVI)

S2 Video Time-lapse observation of wrinkle formation and YFP-Solo localization. MCF10A cells were transfected with YFP-Solo and cultured on a thin Matrigel-coated silicone substrate for 24 h. Red arrowheads on the first frame indicate accumulation of Solo along the wrinkles. Frames were acquired every 5 min for 2.5 h and are displayed at 4 frames/s. Scale bar, 20 μm. Related to S3A Fig, YFP-Solo.
(AVI)
