# Supplementary figures and images for: Solo, a RhoA-targeting guanine nucleotide exchange factor, is critical for hemidesmosome formation and acinar development in epithelial cells
Source: PLoS One. 2018 Apr 19;13(4):e0195124. doi: 10.1371/journal.pone.0195124 (PMC5909619; doi:10.1371/journal.pone.0195124)

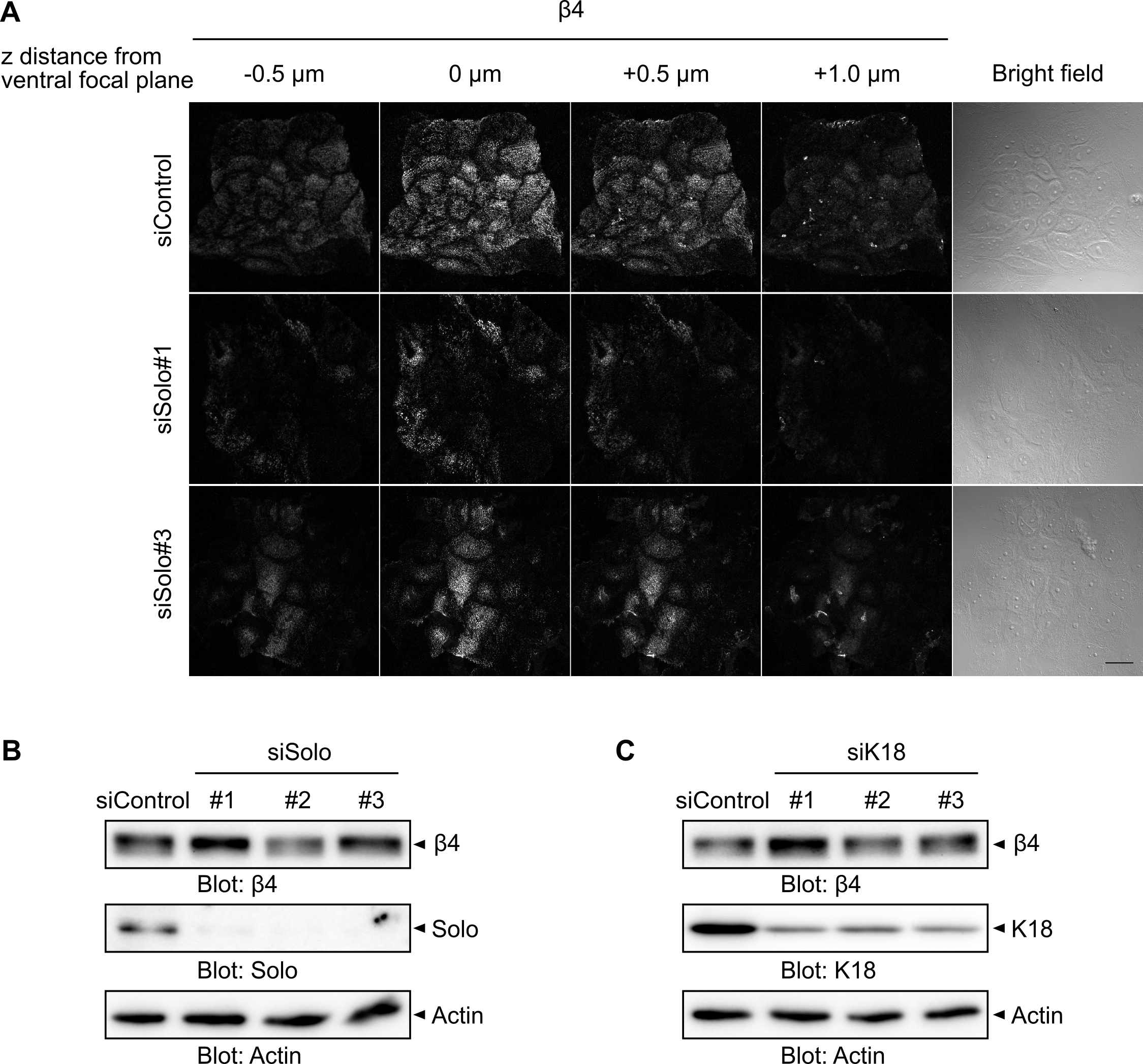

Supplement: S1 Fig — (A) Effect of Solo knockdown on the ventral localization of β4. MCF10A cells were seeded on a thin Matrigel-coated coverslip, transfected with control or Solo-targeting siRNAs, and cultured for 48 h. Cells were then fixed and permeabilized with cold methanol and β4 was stained. The slice image was obtained at the ventral surface where the most abundant and clearest β4 signals were observed, and the additional images were taken every 0.5 μm to the dorsal side (2 slices) and to the ventral side (1 slice) using a confocal microscopy. Scale bar, 20 μm. (B and C) Effect of Solo knockdown (B) and effect of K18 knockdown (C) on the β4 protein expression level. MCF10A cells were transfected with control siRNA or Solo- or K18-targeting siRNAs and cultured for 48 h. Cell lysates were analyzed by immunoblotting with indicated antibodies. Actin was used as a loading control. (TIF) [file pone.0195124.s001.tif]

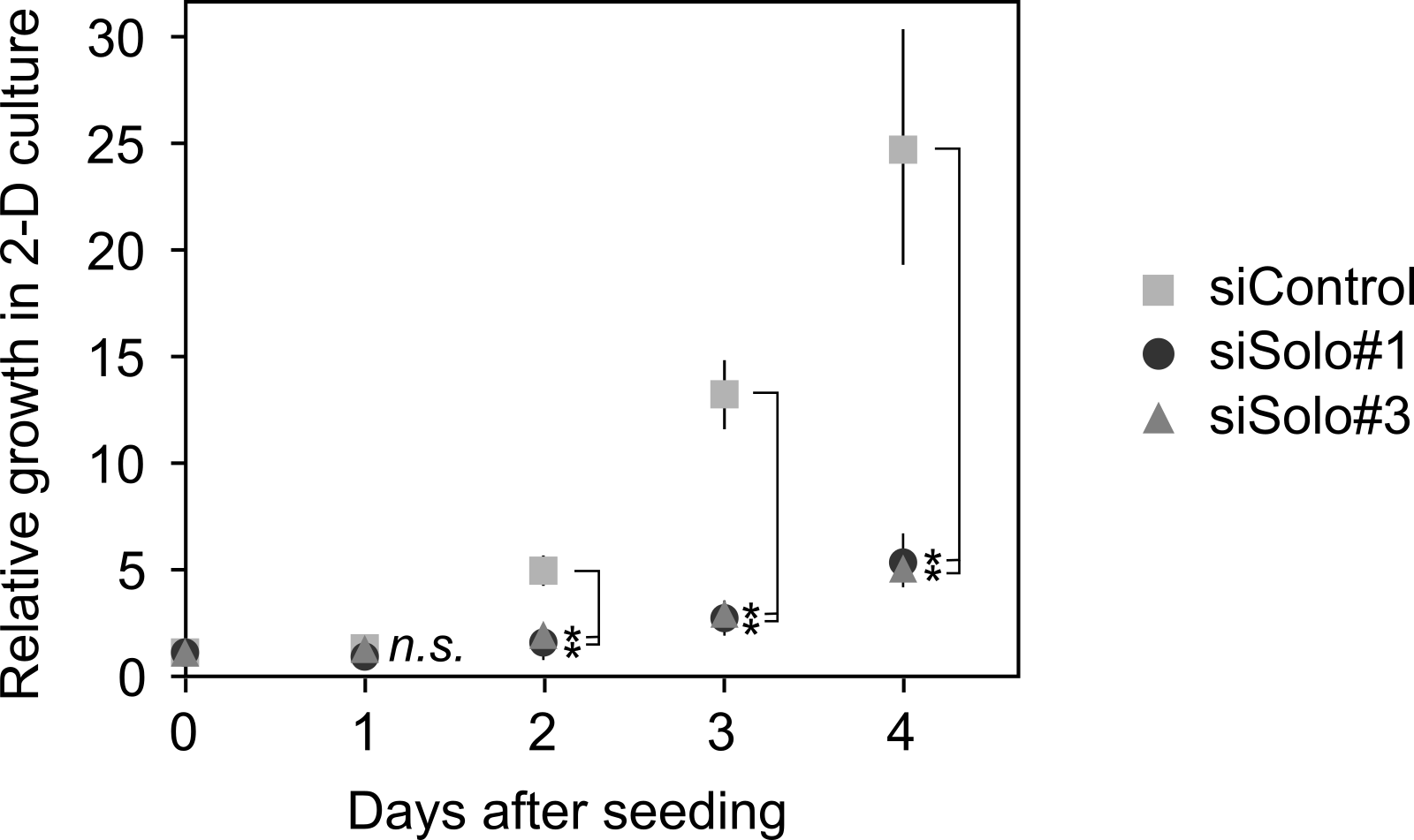

Supplement: S2 Fig — MCF10A cells were transfected with control or Solo-targeting siRNAs, seeded on 35-mm dishes, and then collected. The cell number at indicated days was calculated. Data represent the means ± SD of 3 independent experiments. **P < 0.01 (one-way ANOVA followed by Dunnett's test); n.s., not significant. (TIF) [file pone.0195124.s002.tif]

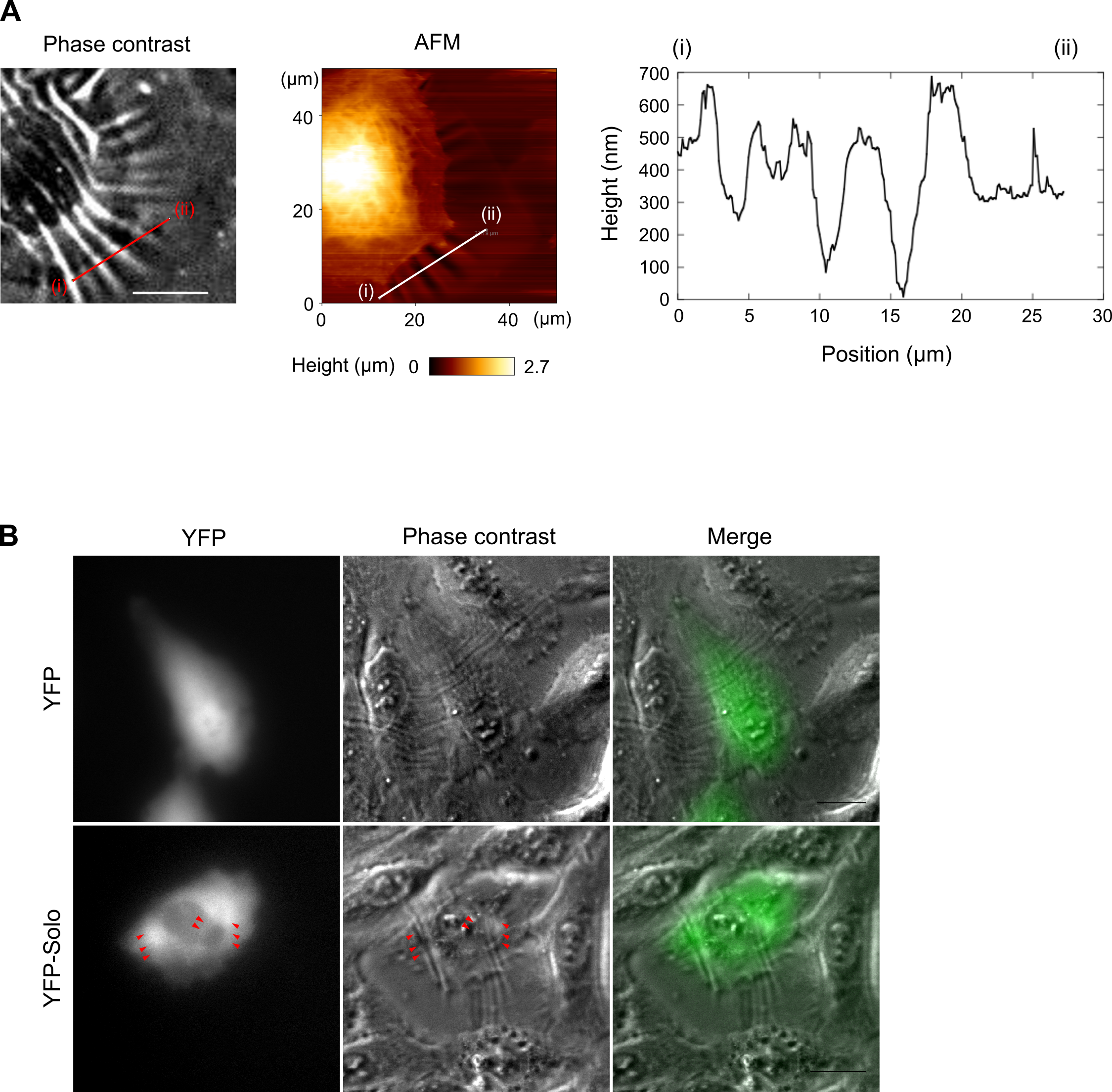

Supplement: S3 Fig — (A) Detailed measurement of the wrinkles on the silicone substrate. Wrinkles generated by a single cell were simultaneously observed by phase-contrast and atomic force microscopies to evaluate the height of the wrinkles along line (i)-(ii). Scale bar, 20 μm. (B) Wrinkle formation assay. MCF10A cells were transfected with YFP or YFP-Solo, seeded on a thin Matrigel-coated silicone substrate, and cultured for 24 h. Time-lapse fluorescence images of YFP (green) and phase-contrast images were acquired every 5 min for 2.5 h (see Supplemental S1 and S2 Videos). Red arrowheads indicate accumulation of Solo along the wrinkles. Scale bar, 20 μm. (TIF) [file pone.0195124.s003.tif]
